# Nipah Virus C Protein Recruits Tsg101 to Promote the Efficient Release of Virus in an ESCRT-Dependent Pathway

**DOI:** 10.1371/journal.ppat.1005659

**Published:** 2016-05-20

**Authors:** Arnold Park, Tatyana Yun, Frederic Vigant, Olivier Pernet, Sohui T. Won, Brian E. Dawes, Wojciech Bartkowski, Alexander N. Freiberg, Benhur Lee

**Affiliations:** 1 Department of Microbiology, Immunology and Molecular Genetics, David Geffen School of Medicine at the University of California-Los Angeles, Los Angeles, California, United States of America; 2 Department of Microbiology, Icahn School of Medicine at Mount Sinai, New York, New York, United States of America; 3 Department of Pathology, University of Texas Medical Branch, Galveston, Texas, United States of America; 4 Center for Biodefense and Emerging Infectious Diseases, University of Texas Medical Branch, Galveston, Texas, United States of America; 5 Institute for Human Infections and Immunity, University of Texas Medical Branch, Galveston, Texas, United States of America; Northwestern University, UNITED STATES

## Abstract

The budding of Nipah virus, a deadly member of the *Henipavirus* genus within the *Paramyxoviridae*, has been thought to be independent of the host ESCRT pathway, which is critical for the budding of many enveloped viruses. This conclusion was based on the budding properties of the virus matrix protein in the absence of other virus components. Here, we find that the virus C protein, which was previously investigated for its role in antagonism of innate immunity, recruits the ESCRT pathway to promote efficient virus release. Inhibition of ESCRT or depletion of the ESCRT factor Tsg101 abrogates the C enhancement of matrix budding and impairs live Nipah virus release. Further, despite the low sequence homology of the C proteins of known henipaviruses, they all enhance the budding of their cognate matrix proteins, suggesting a conserved and previously unknown function for the henipavirus C proteins.

## Author Summary

Nipah virus is a deadly pathogen (40–100% mortality) that has yearly outbreaks in Southeast Asia, resulting from spillover from its natural fruit bat reservoir. The viral C protein is one of only nine virus proteins, but its role in promoting virus replication is not fully understood. Here, we found that the C protein promotes the efficient release of budding Nipah virus from infected cells. It does so by recruiting an essential factor in the host ESCRT complex, Tsg101. The ESCRT complex has well-characterized functions in mediating membrane pinching off events that resemble virus budding. Further, we found that the C proteins of related viruses within the same genus (*Henipavirus*) also promote virus budding, suggesting that this previously unknown function of the henipavirus C proteins is conserved. This work illuminates the basic biology of henipaviruses with significant outbreak and public health concern, and opens the door to further lines of inquiry.

## Introduction

The host ESCRT (endosomal sorting complex required for transport) pathway catalyzes cellular membrane scission events, such as multivesicular body formation and cellular abscission, in which the cytoplasm has access to the inside of the constricting membrane neck [1,2]. Many enveloped viruses have co-opted the ESCRT pathway to catalyze the membrane scission required to pinch off and release budding virions [3]. HIV-1, for example, recruits the ESCRT pathway via specific motifs on the virus Gag protein, and inhibition of ESCRT prevents HIV-1 release from the cell surface [4–6]. Influenza A virus (IAV) is a rare example of an enveloped virus with a delineated ESCRT-independent budding pathway, in which IAV encodes its own factor to catalyze membrane scission [7].

Nipah virus (NiV), a deadly zoonotic virus within the *Henipavirus* genus of the *Paramyxoviridae* family, has been thought to have an ESCRT-independent budding pathway. The Nipah matrix protein (NiV-M) has the central role in virus assembly and budding [8,9]. The budding of NiV-M, which is released from cells in virus-like particles (VLPs) when expressed on its own, was not affected by inhibition of ESCRT [10]. Although two motifs in NiV-M have been suggested as potential late domains (ESCRT-dependent motifs that trap budding at a “late” step when disrupted), no evidence that they act as late domains in the context of NiV-M was presented [10,11]. As this lack of evidence for a role for ESCRT in NiV budding was based on NiV-M VLP release in the absence of other viral components, we wondered if NiV budding might be ESCRT-dependent in the context of the full virus.

Inhibition of ESCRT resulted in a significant decrease in released NiV titers of several logs, indicating that in the context of live virus replication, NiV has an ESCRT-dependent budding pathway. We then investigated whether viral components other than NiV-M might be responsible for this reliance on ESCRT. To our surprise, we found that the NiV C protein, an accessory factor previously thought to solely play a role in counteracting innate immunity, enhanced NiV-M budding in an ESCRT-dependent manner. This finding was reminiscent of previous findings that the C protein of Sendai virus (SeV, a related paramyxovirus in the *Respirovirus* genus) enhanced SeV-M release in a manner dependent on the ESCRT factor Alix [12–14]. However, depletion of Alix or inhibition of ESCRT was not found to affect the budding of live SeV [15], making the functional significance of these findings regarding SeV-C unclear.

Interestingly, a large segment of NiV-C aligned with the essential host ESCRT factor Vps28. We found that like Vps28, NiV-C interacted directly with the ESCRT factor Tsg101, which was required for the NiV-C enhancement of budding as well as the efficient release of live NiV. Finally, the C protein of other known henipaviruses also enhanced the budding of their cognate matrix proteins, suggesting a conserved role for the henipavirus C protein in the budding process.

## Results

### Nipah virus budding is ESCRT-dependent

**Fig 1:**
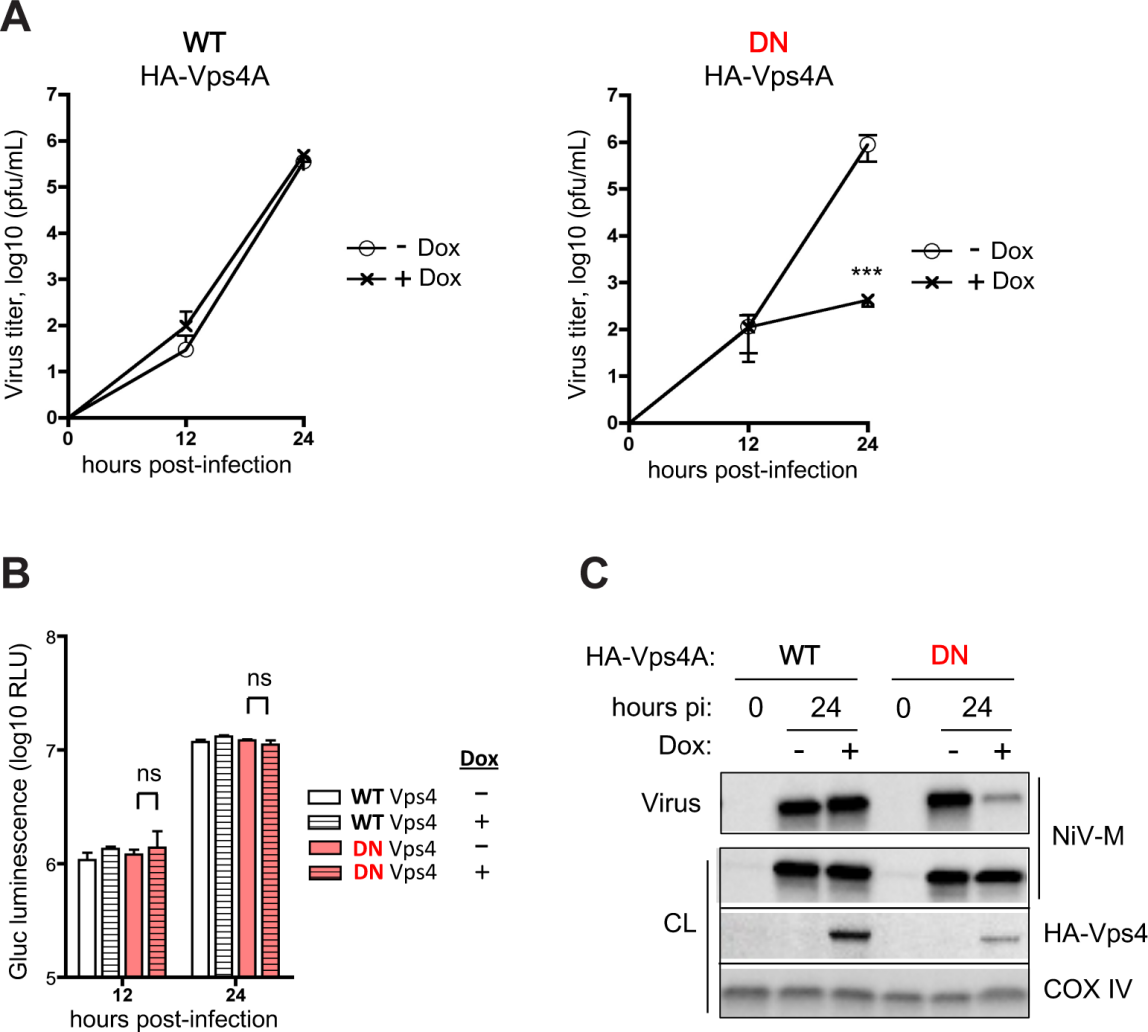
Nipah virus budding is ESCRT-dependent. **(A)** Wild-type (WT) and dominant-negative (DN) Vps4A-inducible 293 cells were infected with *Gaussia* luciferase-expressing NiV at a multiplicity of infection (MOI) of 2. Cells were induced with doxycycline (dox) post-infection, and viral titers in the supernatant were determined at the indicated time points. Error bars represent standard deviations for 3 replicates. Induction of DN Vps4A significantly inhibits the release of NiV at 24 hours post-infection (hpi). ***, p<0.001, two-way ANOVA followed by Bonferroni posttests. **(B)** Induction of WT or DN Vps4A did not affect *Gaussia* luciferase (Gluc) production, indicating unimpaired virus protein production. ns, not significant, two-way ANOVA followed by Bonferroni posttests. **(C)** Vps4A-inducible 293 cells were infected with wild-type NiV at MOI 2, and Vps4A (WT vs. DN) was induced (+Dox) or not (-Dox) immediately after infection as in Fig 1A. At 24 hpi, cell lysates (CL) were collected, and virions were ultracentrifuged through 20% sucrose and resuspended in Laemmli SDS sample buffer for Western analysis. The 0 hpi time point represents the last wash after removal of the inoculum to determine the background level of residual virus. Induction of DN Vps4A clearly resulted in less virions (NiV) released into the supernatant.]

The classical test for ESCRT-dependence is sensitivity to a dominant-negative Vps4, the indispensable ATPase that drives the scission process [1,3]. We created 293 stable cell lines with inducible overexpression of either wild-type (WT) or dominant-negative (DN) Vps4A (S1 Fig). To interrogate the involvement of the ESCRT pathway in the budding of live NiV, we infected the Vps4A-inducible 293 cell lines with recombinant NiV expressing a secreted *Gaussia* luciferase reporter [16–18]. WT or DN Vps4A was induced post-infection, and not before, to avoid any potential effect of Vps4A overexpression on the infection event itself. While induction of WT Vps4A did not affect viral titers released into the supernatant at 12 and 24 hours post-infection (hpi), induction of DN Vps4A reduced such titers by over 3 logs at 24 hpi (Fig 1A). Notably, neither WT nor DN Vps4A affected the amount of *Gaussia* luciferase reporter secreted into the infected cell supernatant, indicating that the defect in released viral titers was not due to a deleterious effect on virus protein production *per se* (Fig 1B). Furthermore, when we infected the inducible cell lines with wild-type non-recombinant NiV and compared the amount of NiV-M found in pelleted virions by Western analysis, we found that the induction of DN but not WT Vps4A resulted in a marked reduction in NiV-M release (Fig 1C). Altogether, our results suggest that virion production and release in the context of live NiV replication is an ESCRT-dependent process that is sensitive to DN Vps4A inhibition.

### NiV-M budding is enhanced by NiV-C in an ESCRT-dependent manner

**Fig 2:**
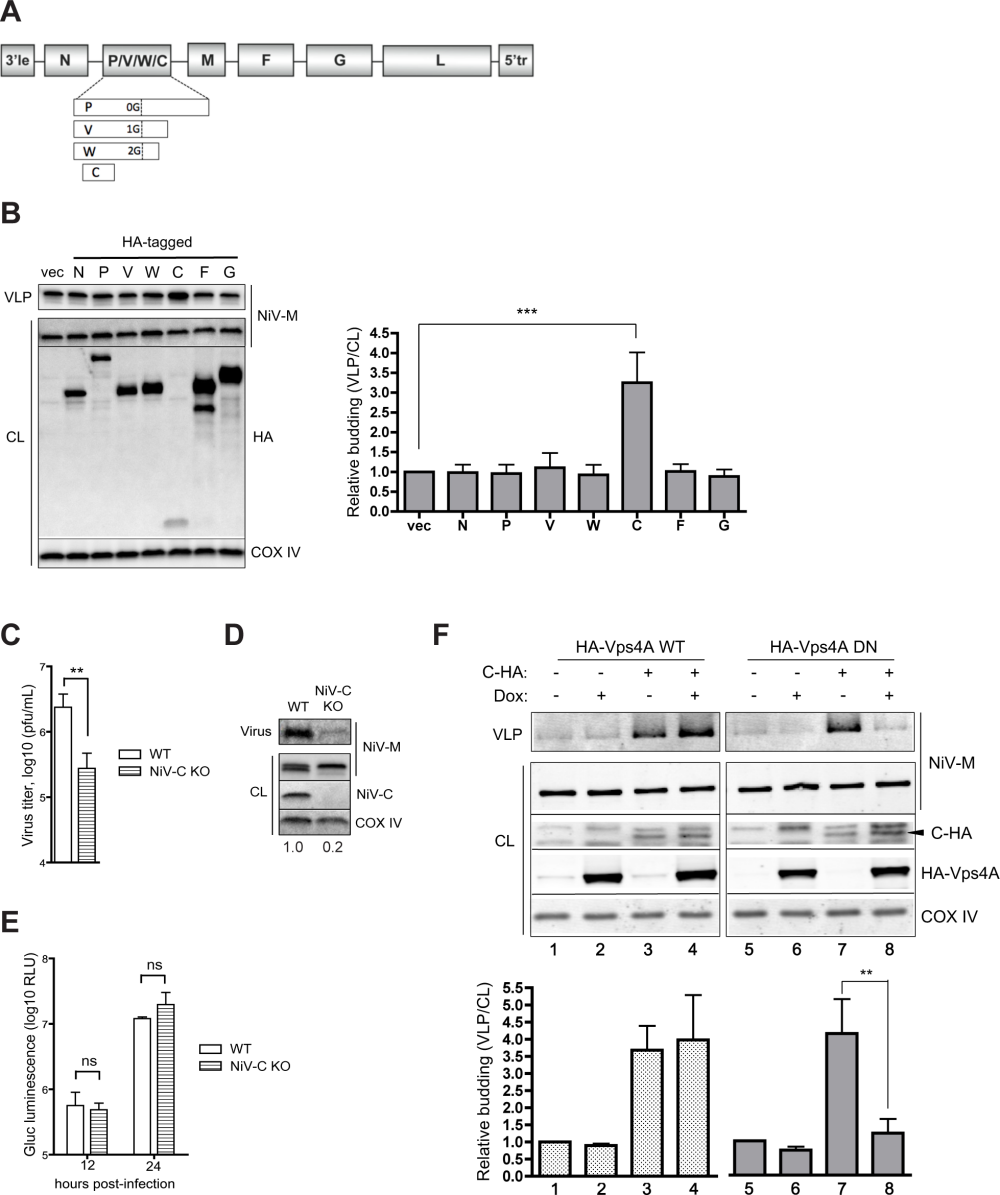
NiV-M budding is enhanced by NiV-C in an ESCRT-dependent manner. **(A)** A schematic of the NiV genome is shown, with the NiV genes arrayed between the genomic termini, the 3’ leader (3’le) and 5’ trailer (5’tr). NiV-V and -W are produced by the stochastic inclusion of additional G nucleotides in the P mRNA via polymerase stuttering (as indicated in the schematic), thus creating a frameshift at that juncture, while NiV-C is produced off an alternate reading frame. **(B)** NiV-M was co-transfected with vector only or with HA-tagged NiV-derived gene products into 293T cells, and relative virus-like particle budding was determined as described in Materials and Methods. COX IV is shown as the cell lysate loading control. Error bars represent standard deviations for 3 independent experiments. Only NiV-C had a significant effect on M budding relative to the vector control. ***, p<0.001, one-way ANOVA followed by Bonferroni posttests. **(C)** CCL-81 Vero cells were infected with Gluc-expressing WT or NiV-C knockout NiV at MOI 2. Virus titers were determined from supernatant collected at 24 hpi. Error bars represent standard deviations for 3 replicates. **, p<0.01, 2-tailed Student’s t-test. **(D)** At 24 hpi, cell lysates (CL) were collected, and virions were ultracentrifuged through 20% sucrose and resuspended in Laemmli SDS sample buffer for Western analysis. Normalized relative budding values are shown below the blot. **(E)** Knockout of NiV-C did not affect Gluc production, indicating unimpaired protein production. ns, not significant, two-way ANOVA followed by Bonferroni posttests. **(F)** Vps4A-inducible 293 cells were transfected with NiV-M and with or without NiV-C-HA as indicated. At 4 hours post-transfection, media was changed with or without doxycycline. Error bars represent standard deviations for 3 independent experiments. **, p<0.01, one-way ANOVA followed by Bonferroni posttests. Only NiV-C enhancement of NiV-M-mediated VLP budding was significantly affected by induction of DN Vps4A.

NiV-M budding in the absence of other virus components is ESCRT-independent and insensitive to DN-Vps4A inhibition [10]. However, since live NiV budding appears to be ESCRT-dependent, and is sensitive to DN Vps4A inhibition, we wondered what viral component(s) might promote M-mediated virus budding in an ESCRT-dependent pathway. Previous studies suggested that co-expression of NiV nucleocapsid protein (N), fusion protein (F), or attachment protein (G) did not affect matrix release [9,11]; however, other NiV proteins such as the phosphoprotein (P), V protein (V), W protein (W), and C protein (C) (Fig 2A) were not tested for a potential role in budding, perhaps due to their previously characterized roles in virus transcription and/or antagonism of innate immunity.

We co-transfected NiV-N, -P, -V, -W, -C, -F, and -G with NiV-M in 293T cells in a VLP budding assay. NiV-M VLPs released in the supernatant were pelleted through a 20% sucrose cushion, and relative amounts of M in VLPs versus cell lysates were compared by quantitative Western blotting. Consistent with previous reports, co-expression of NiV-N, -F, and -G did not affect the efficiency of M VLP release (Fig 2B). Only co-expression of NiV-C significantly enhanced M VLP release (Fig 2B), suggesting a role for C protein in the NiV budding pathway. Importantly, NiV-C and NiV-M expression from our transient transfection studies was equal to or lower than the natural level of expression observed in live NiV infection when comparing equivalent conditions and time points (S2 Fig). These data underscore the physiological relevance of our findings.

We reasoned that if C protein had a role in budding, knockout of C protein from the live virus should result in decreased virus release. We mutated the Gluc-expressing NiV to delete expression of NiV-C without affecting expression of the overlapping NiV-P ORF, as done previously [19–22]. C-deficient NiV had decreased virus release as determined by infectious titers (Fig 2C) as well as Western analysis of physical virion production (Fig 2D), despite having equivalent virus protein production as determined by the Gluc reporter (Fig 2E).

Finally, to determine whether the C enhancement of NiV-M budding was dependent on ESCRT, we performed the M VLP budding assay in the Vps4A-inducible 293 cells. While NiV-C enhancement of M release was not affected by induction of WT Vps4A expression (Fig 2F, compare lanes 3 and 4), it was abrogated by DN Vps4A (Fig 2F, compare lanes 7 and 8). Altogether, our data suggest that NiV-C enhances NiV-M budding in an ESCRT-dependent manner.

### NiV-C interacts with NiV-M to promote budding

The NiV C protein has mainly been studied for its role in antagonism of innate immunity [20–23], although there is evidence that NiV-C has other functions in NiV replication. For example, while C-deficient NiVs, created through reverse genetics, induce a more robust IFN-β response and are attenuated both *in vitro* and *in vivo*, C-deficient NiVs are also unexpectedly attenuated in IFN-α/β -deficient Vero cells (1–2 logs reduction in viral titers compared to wild-type NiV) (Fig 2C) [19,20,22]. The latter suggests that the C protein has additional proviral function(s) that are independent of its immune antagonism activities. Our data suggest that C protein enhancement of NiV-M budding may be one such function.

**Fig 3:**
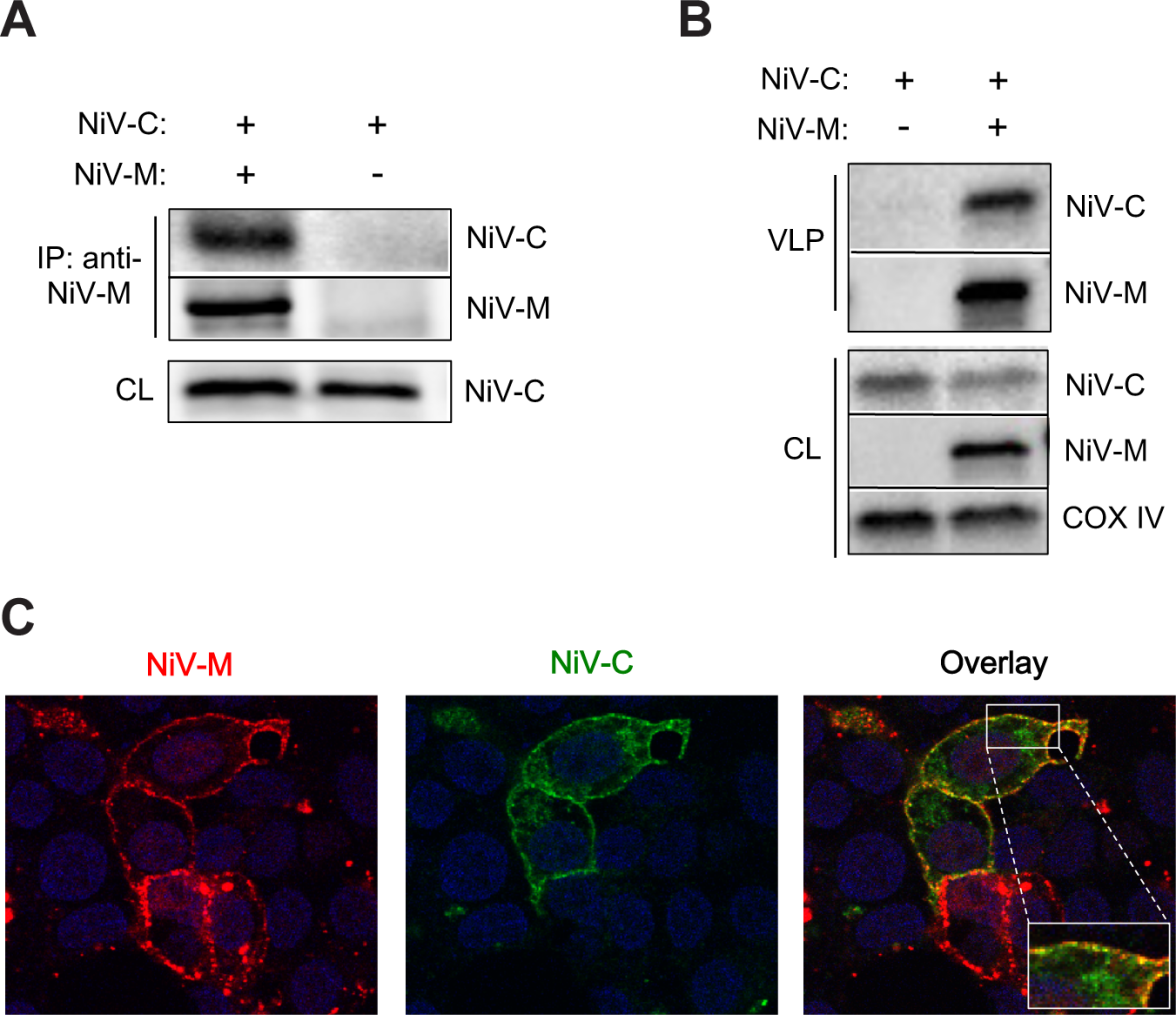
NiV-C interacts with NiV-M to promote budding. **(A)** NiV-C-HA and NiV-M were transfected as indicated in 293T cells, and lysates were immunoprecipitated with anti-NiV-M as described in Materials and Methods. NiV-C was detected with anti-HA. **(B)** NiV-C-HA and NiV-M were transfected as indicated in 293T cells. NiV-C was detected with anti-HA. NiV-C is only detected in VLPs in the presence of NiV-M VLP budding. **(C)** NiV-M and NiV-C-HA were co-transfected in 293T cells and detected by confocal microscopy as described in Materials and Methods. DAPI (blue) illustrates the nuclei. A representative Z-slice is shown.

Thus, we asked whether and how the NiV-C protein might interact with NiV-M to promote budding. We found that NiV-C and NiV-M interact by co-immunoprecipitation (Fig 3A), that NiV-C does not bud by itself but is efficiently incorporated into M VLPs (Fig 3B), and that NiV-C and NiV-M are co-localized at the cell surface as revealed by confocal microscopy (Fig 3C). Our results are consistent with a previous report that C protein is present in NiV virions [24] and suggest that C protein may directly interface with NiV-M at the cell surface to promote M VLP release.

### C enhancement requires a cluster of residues conserved between NiV-C and host factor Vps28

**Fig 4:**
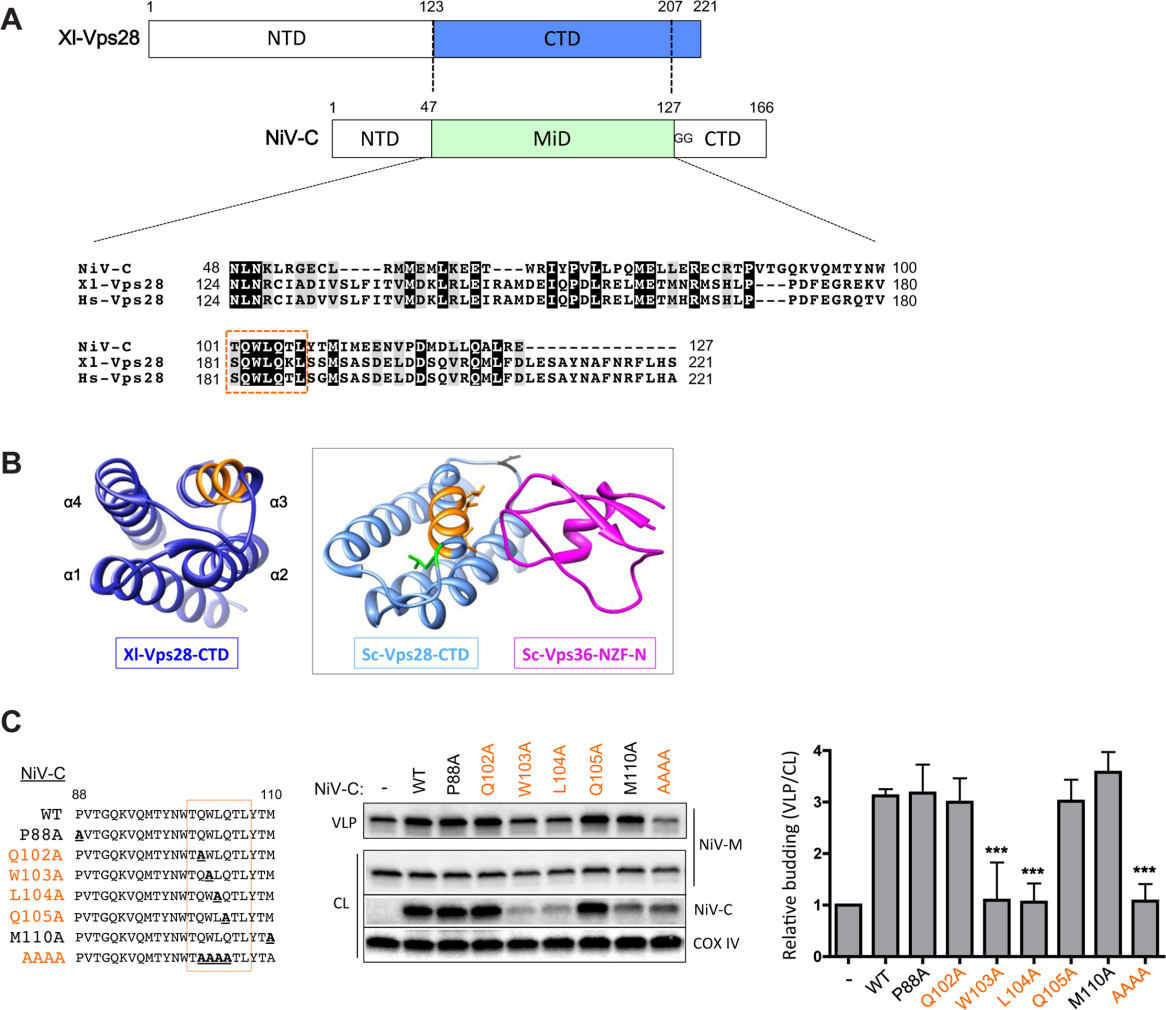
C enhancement requires a cluster of residues conserved between NiV-C and host factor Vps28. **(A)** A structural homology modeling search of NiV-C in the Phyre2 server (Imperial College, London) revealed an alignment with the C-terminal domain (CTD) of Vps28 of *X*. *laevis* (Xl-Vps28). Based on the alignment, we defined a N-terminal domain (NTD), middle domain (MiD), and C-terminal domain (CTD) for NiV-C. The presence of a double glycine (GG) immediately following NiV-C-MiD is suggestive of a linker between domains. The sequence alignment was created in Clustal Omega, with illustration via BOXSHADE, with conserved (black) and similar (grey) residues highlighted. The CTD of human Vps28 (Hs-Vps28) is included in the alignment for comparison. A nearly identical stretch of 7 amino acids between NiV-C and Hs-Vps28 is boxed in orange. **(B)** *Left*, the *X*. *laevis* Vps28-CTD structure (dark blue) is shown, with the stretch of 7 amino acids indicated in the sequence alignment in Fig 4A highlighted in orange in their homologous positions [26] (PDB 2J9W, displayed in Chimera). The N- to C-terminal helices of the four-helix bundle are labeled as α1–4. *Right and boxed*, rotated view of *S*. *cerevisiae* Vps28-CTD (Sc-Vps28-CTD, light blue) and Vps36 Npl4 zinc-finger N-terminal domain (Sc-Vps36-NZF-N, magenta), shown in their known structural interaction [26] (PDB 2J9U). The same stretch of 7 amino acids is also highlighted here in orange in their homologous positions (residues 203–209 in Sc-Vps28) in the Sc-Vps28-CTD structure. Residues I203, I206, and V207 within this stretch are functionally important in the yeast Vps28-Vps36 interface [26] and their side chains are illustrated. Black and green highlight in the Sc-Vps28-CTD structure illustrate residues discussed in Fig 4C (see following). **(C)** Alanine mutagenesis in full length NiV-C-HA was performed as shown. The positions of P88 and M110 in their homologous positions in Sc-Vps28-CTD are shown in black and green highlight (D194 in black and L212 in green, respectively) in Fig 4B. P88 is within the loop between helices 2 and 3, which differs by 2 residues in length between metazoan and yeast Vps28. Due to differing alignments in this region, the closest residue in yeast Vps28 (D194) in most considered alignments was chosen for comparison [26–28]. Mutation of W103 and L104 within the highlighted stretch of residues selectively abrogates the ability of NiV-C to enhance NiV-M budding. Error bars represent standard deviations for 3 independent experiments. ***, p<0.001, one-way ANOVA followed by Bonferroni posttests.

To gain further insight into the mechanism of NiV-C enhancement of M budding, we performed a structural homology modeling search of NiV-C using the Phyre2 program [25]. Interestingly, a large middle segment (MiD) of the C protein aligned to the C-terminal domain (CTD) of the host factor Vps28, an essential component of the ESCRT pathway (Fig 4A).

Vps28, a component of the ESCRT-I subcomplex, plays an adaptor role between ESCRT-I and downstream factors, particularly the ESCRT-II subcomplex [1,29]. In yeast, the N-terminal domain of Vps28 interacts directly with Vps23 (human Tsg101) in ESCRT-I, whereas the C-terminal domain interacts directly with Vps36 in ESCRT-II. Since the MiD of NiV-C aligned with the C-terminal domain of Vps28, we wondered if NiV-C might also analogously interact with human Vps36 (hVps36). However, we could not detect specific co-immunoprecipitation of endogenous or overexpressed hVps36 with either NiV-C or human Vps28 (hVps28). This is perhaps not surprising as the ESCRT components between yeast and humans, though highly conserved, are not completely orthologous. For example, while hVps28 and hVps36 can be found associated with other factors in a complex, the interaction of recombinant hVps28 and hVps36, unlike their yeast counterparts, could not be detected in the absence of these other factors [30]. Indeed, hVps28 and hVps36 likely interact via a different, yet to be characterized interface, as charged side chains in metazoan Vps28 not present in the homologous yeast Vps28 would disrupt the known interface between yeast Vps28 and Vps36. Furthermore, metazoan Vps36 also lacks the zinc-finger domain present in yeast that interacts with Vps28 [1,26].

The NiV-C-aligned C-terminal domain of Vps28 forms a conserved four α-helix bundle (Fig 4B) [26,28]. Despite the abovementioned differences, close inspection of the alignment between NiV-C and hVps28 revealed a striking stretch of nearly identical amino acids between NiV-C and hVps28 (Fig 4A, orange box) that is predicted to be in the 3rd α-helix, which in the homologous yeast (*S*. *cerevisiae*) structure lies at the interface between Vps28 and Vps36 [26] (Fig 4B). Since NiV-C appears to mimic this stretch of residues in Vps28, we hypothesized that this Vps28 interface is functionally conserved in the ESCRT recruitment machinery even if the specific ESCRT components recruited by yeast and human Vps28 might actually differ. Our hypothesis predicted that disruption of this conserved, strategically located stretch of residues in NiV-C might also disrupt the NiV-C function of enhancing the budding of NiV-M.

To interrogate our hypothesis, we mutated the central portion of this conserved stretch in NiV-C to alanines, and compared these mutants to alanine mutations in neighboring residues also conserved in the alignment between NiV-C and hVps28, but not predicted to be functional Vps28-Vps36 interface residues in their homologous positions in yeast Vps28 (Fig 4B and 4C). Mutagenesis of W103 and L104 within this central conserved stretch, but not the neighboring residues (P88 and M110), significantly reduced the NiV-C enhancement of NiV-M budding (Fig 4C). Although mutagenesis of W103 and L104 led to lower expression of NiV-C, the M110A mutant, which also had lower NiV-C expression, continued to enhance NiV-M budding as well as WT NiV-C. This result is consistent with the possibility that NiV-C-mediated recruitment of ESCRT-II is important for the budding role of C protein.

### NiV-C has a direct interaction with Tsg101

**Fig 5:**
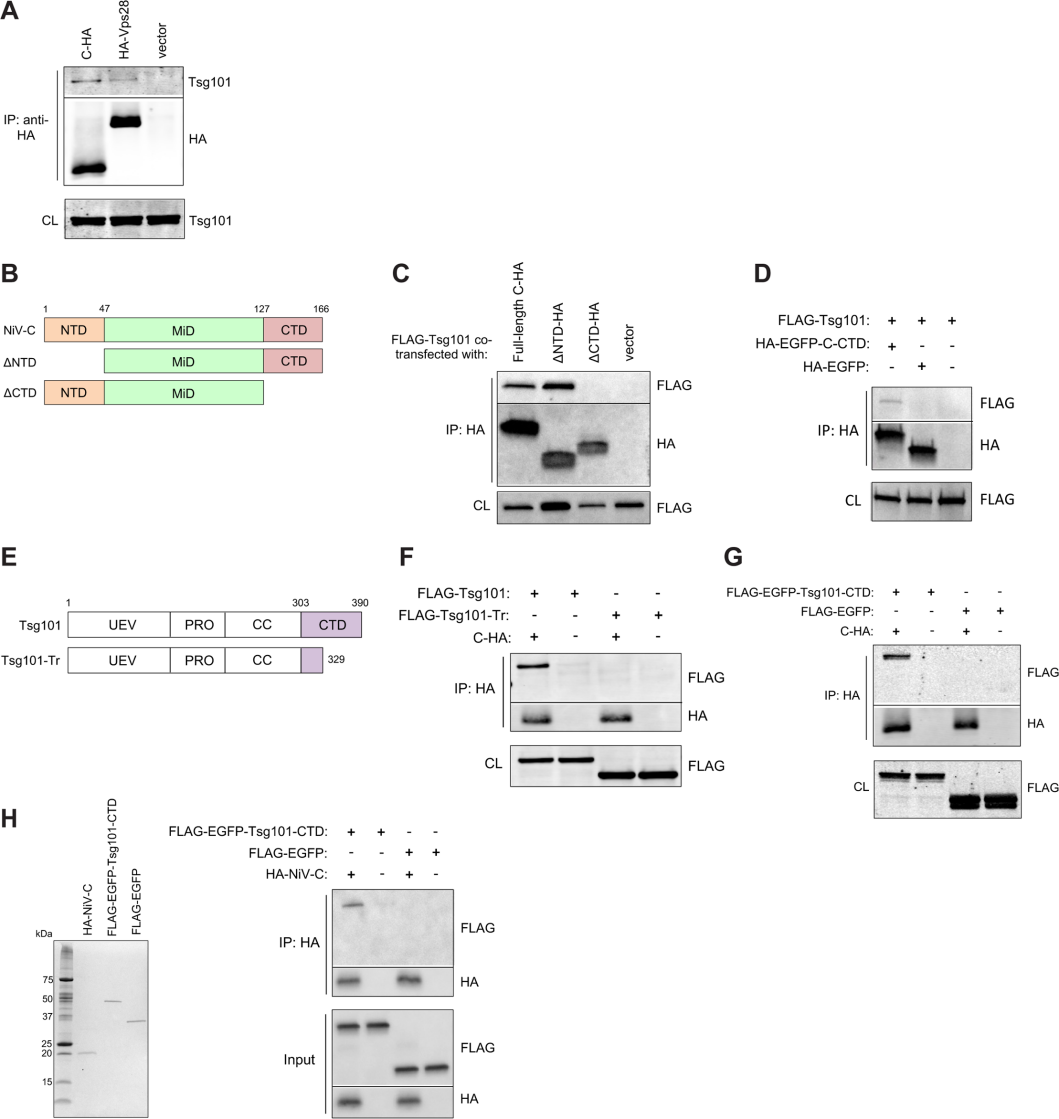
NiV-C has a direct interaction with Tsg101. **(A)** HA-tagged NiV-C co-immunoprecipitates endogenous Tsg101 from 293T cell lysates, as does HA-tagged Vps28, a known cellular interacting partner of Tsg101. **(B)** NiV-C was truncated as indicated, with all constructs retaining a C-terminal HA tag. **(C)** Loss of the CTD of NiV-C specifically leads to loss of pulldown of Tsg101. **(D)** A fusion of EGFP and NiV-C-CTD (HA-EGFP-C-CTD) pulls down Tsg101, whereas EGFP alone does not. **(E)** The C terminal domain (CTD) of Tsg101 was defined as in [31]. FLAG-Tsg101 was truncated as indicated. UEV, ubiquitin E2 variant domain; PRO, proline-rich domain; CC, coiled-coil region; and CTD, C-terminal domain. **(F)** Truncation of the CTD of Tsg101 results in loss of co-immunoprecipitation with NiV-C. **(G)** A fusion of EGFP and the full CTD (amino acids 303–390) of Tsg101 (FLAG-EGFP-Tsg101-CTD) co-immunoprecipitates with NiV-C, whereas EGFP alone does not. **(H)** *Left panel*, Coomassie-stained gel showing recombinant proteins (100 ng each lane) purified from *E*. *coli* as described in Materials and Methods. *Right panel*, Western analysis with repeat of Fig 5G except with purified recombinant proteins, showing that the interaction between NiV-C and Tsg101-CTD is direct.

The alignment of NiV-C with Vps28 suggested that C protein might also interact with the other known interacting partner of Vps28, the ESCRT-I factor Tsg101. Indeed, like hVps28, NiV-C immunoprecipitated endogenous Tsg101 (Fig 5A). To define the portion of NiV-C responsible for this interaction, we performed truncations of NiV-C at the N- (ΔNTD) and C-terminal (ΔCTD) boundaries of the aligned middle domain (MiD) (Fig 5B). A double glycine immediately following NiV-C-MiD, perhaps representing a flexible linker between domains (Fig 4A), allowed for a natural demarcation for making the ΔCTD truncation mutant. Loss of the C-terminal domain (ΔCTD) abrogated the interaction of NiV-C with Tsg101 (Fig 5C). Furthermore, the CTD of NiV-C fused to EGFP, but not EGFP alone, also interacted with Tsg101, indicating that the CTD of NiV-C was both necessary and sufficient for the interaction (Fig 5D).

We then determined the corresponding NiV-C-interacting domain on Tsg101. Vps28 is known to interact with the C-terminal domain of Tsg101 (Tsg101-CTD) [31–33]. Following the hypothesis that NiV-C mimics the interactions of Vps28, we wondered whether NiV-C might also interact with Tsg101-CTD, analogous to Vps28. Truncation of Tsg101-CTD led to loss of interaction with NiV-C (Fig 5E and 5F). Conversely, Tsg101-CTD fused to EGFP, but not EGFP alone, was sufficient to bring down NiV-C (Fig 5G). These results indicated that Tsg101-CTD was both necessary and sufficient for the interaction.

Finally, to determine if the interaction between NiV-C and Tsg101 was direct, we purified recombinant full-length NiV-C, the EGFP-Tsg101-CTD fusion, and EGFP alone from *E*. *coli* (Fig 5H, left panel). As in Fig 5G, but with the purified recombinant proteins, we showed that NiV-C co-immunoprecipitated EGFP-Tsg101-CTD but not EGFP alone, suggesting that the interaction between NiV-C and Tsg101 was indeed direct (Fig 5H, right panel). Altogether, our results show that NiV-C-CTD and Tsg101-CTD, the C-terminal domains of NiV-C and Tsg101, respectively, interact directly without the need for any intermediary host factors.

### Tsg101 is required for the NiV-C enhancement of budding and efficient release of live NiV

**Fig 6:**
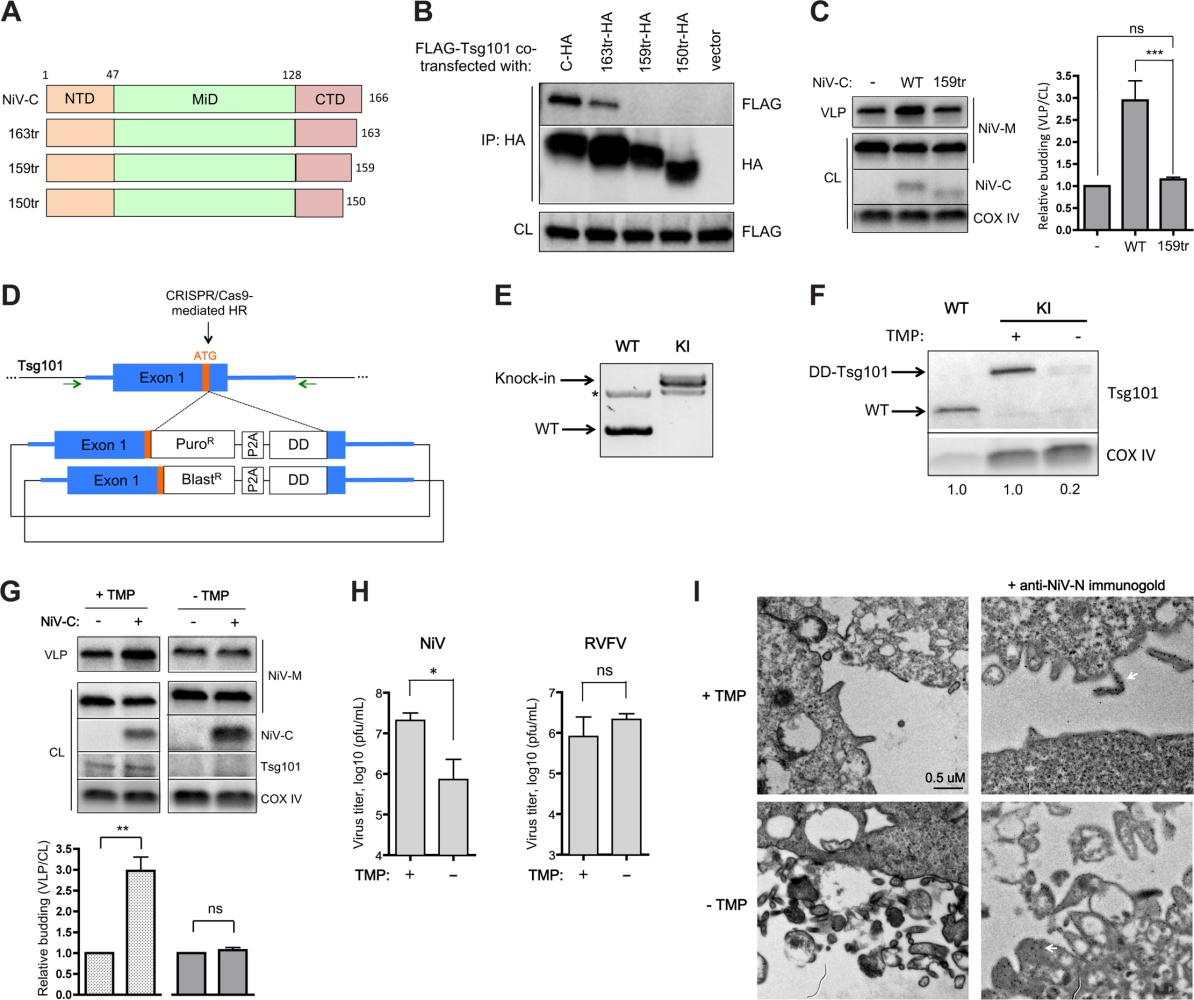
Tsg101 is required for the NiV-C enhancement of budding and efficient release of live NiV. **(A)** NiV-C was minimally truncated at its C-terminus as indicated, with all constructs retaining a C-terminal HA tag. **(B)** Truncation of only 7 residues (159tr) from NiV-C results in loss of interaction with Tsg101. **(C)** This truncation mutant no longer enhances NiV-M budding. Error bars represent standard deviations for 3 independent experiments. ns, not significant; ***, p<0.001, one-way ANOVA followed by Bonferroni posttests. **(D)** Schematic of strategy to insert a destabilization domain (DD) tag onto all endogenous copies of Tsg101, via CRISPR/Cas9-mediated homologous recombination (HR). Exon 1 of Tsg101 in the human genome is shown, with the start codon highlighted in orange. Light blue highlight represents sequence used in the homology arms in the donor constructs. Once integrated into the genome, antibiotic resistance is driven off of the endogenous promoter, with the P2A ribosomal skipping sequence allowing the DD-Tsg101 fusion product to be translated separately. Green arrows indicate position of genotyping primers used for Fig 6E. **(E)** Genotyping PCR of the single cell-isolated DD-Tsg101 293T clone with complete knock-in (KI), using genome-specific primers (see Fig 6D, green arrows) flanking the homology arms used in the donor constructs. Asterisk represents a background product. The puromycin and blasticidin donors insert 1,182 and 981 bp, respectively, hence the doublet seen in the knock-in PCR product. **(F)** Knock-in DD-Tsg101 cells (KI) were incubated with 10 uM trimethoprim (TMP) (+ TMP) or DMSO vehicle control (- TMP) for 24h, then collected for comparison with parental 293T cells (WT) by Western. The upward shift in molecular weight for DD-Tsg101 is due to the 18 kDa DD tag fusion. The relative expression of Tsg101 indicated below the blot was normalized to the COX IV loading control. **(G)** DD-Tsg101 cells were incubated in 10 uM TMP or DMSO vehicle as described in Materials and Methods before transfection with NiV-M and with or without NiV-C as indicated. Destabilization of DD-Tsg101 by removal of TMP abrogates the NiV-C enhancement of NiV-M budding. Error bars represent standard deviations for 3 independent experiments. ns, not significant; **, p<0.01, 2-tailed Student’s t-test. **(H)** DD-Tsg101 cells in 10 uM TMP or DMSO vehicle were infected with wild-type NiV at MOI 2 or Rift Valley fever virus (RVFV) at MOI 0.2, and supernatant titers were determined at 24 hpi. Error bars represent standard deviations for 3 replicates. ns, not significant; *, p<0.05, 2-tailed Student’s t-test. **(I)** DD-Tsg101 cells in 10 uM TMP or DMSO vehicle were infected with wild-type NiV at MOI 2 and processed for TEM as described in Materials and Methods. All fields are shown at the same magnification. *Right panels*, samples were stained with anti-NiV-N and 15 nm colloidal gold. White arrows show examples of immunogold staining.

Since hVps28 interaction with Tsg101 plays a critical role in the recruitment of ESCRT complexes that lead to membrane scission or budding events, we hypothesized that NiV-C-mediated recruitment of Tsg101 is also essential for its enhancement of NiV-M budding. To test this hypothesis, we identified the minimal truncation in NiV-C-CTD that would result in disruption of interaction with Tsg101, and we then determined whether this mutant would no longer enhance M release. Loss of only seven residues from the C-terminus (159tr) resulted in loss of interaction with Tsg101 (Fig 6A and 6B). This truncation mutant was not able to enhance M release (Fig 6C), consistent with the importance of the interaction with Tsg101. We therefore sought to determine whether depletion of Tsg101 would affect the C enhancement of M release as well.

Tsg101 is essential for cell growth and proliferation, and ablation of Tsg101 results in embryonic lethality in the peri-implantation period [34,35]. As such, it may be difficult to generate a stable Tsg101 knockout cell line. To overcome this barrier, we used a strategy that allowed maintenance of the essential function of Tsg101, and thus cell viability, until knockdown was desired. To do this, we leveraged CRISPR technology to knock-in a destabilization domain tag (DD) onto all endogenous copies of Tsg101 (Fig 6D), similar to a strategy we published previously for the essential gene TCOF1 [36]. In the presence of a stabilizing compound (in this case the antibiotic trimethoprim), the fusion protein is protected from proteasomal degradation, whereas in the absence of compound, the DD tag targets the fusion protein for degradation. Because removal of compound specifically targets only the DD-tagged protein for degradation, one can therefore test the effects of knockdown in an isogenic cell line with minimal off-target effects.

Genotyping PCR confirmed complete knock-in of the DD tag in all loci of single cell-cloned 293T cells (Fig 6E). We confirmed that removal of compound indeed destabilized DD-Tsg101 (Fig 6F) and resulted in impaired cellular growth (S3 Fig), as expected from the loss of the normal cellular functions of Tsg101, Removal of compound and thus depletion of Tsg101 abrogated the C enhancement in the knock-in DD-Tsg101 cells (Fig 6G). This result suggested that Tsg101 is required for the NiV-C enhancement of M release.

Next, we tested whether specific depletion of Tsg101 in the DD-Tsg101 knock-in cells would impair live NiV release. In experiments performed under BSL4 conditions, removal of the stabilizing compound resulted in a significant 1.5 log reduction in released titers in DD-Tsg101 cells (Fig 6H, left panel). In contrast, removal of the compound resulted in no reduction in the release of Rift Valley fever virus (Fig 6H, right panel), a bunyavirus without a matrix protein analog or any known role for ESCRT in its budding pathway [37].

Finally, we examined whether we could detect a defect in the late stages of budding by transmission electron microscopy (TEM). Although depletion of Tsg101 impaired NiV release, the virus was still able to bud and replicate, albeit at a significantly lower efficiency. We therefore expected that while TEM might not show clear signs of “trapped” virions as for HIV-1, which cannot bud without ESCRT, it might reveal a more disorganized, inefficient budding process upon depletion of Tsg101. Indeed, Tsg101 depletion resulted in accumulation of cell-associated virions that appeared in many cases to be stuck to each other and to the cell, suggestive of an inefficient budding process (Fig 6I). Paramyxovirus particles are much more pleiomorphic than HIV particles but identification is aided by the immunogold labeling (right panels, Fig 6I).

### The C proteins of other known henipaviruses also enhance the budding of their cognate matrix proteins

**Fig 7:**
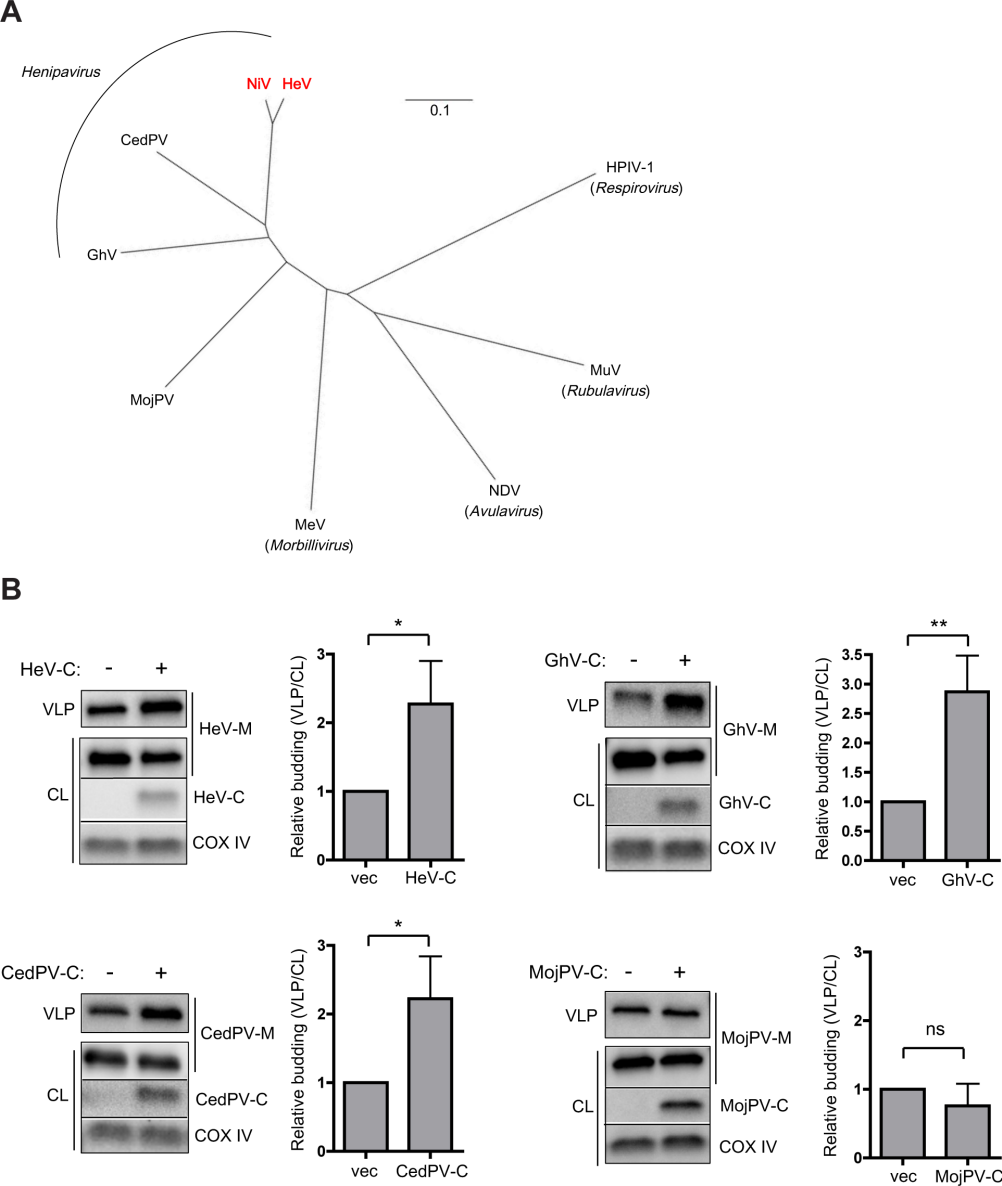
The C proteins of other known henipaviruses also enhance the budding of their cognate matrix proteins. **(A)** Phylogeny (based on the relatively conserved nucleocapsid protein, neighbor-joining tree from Clustal Omega, visualized in FigTree) for the henipaviruses as well as the “henipa-like” Mojiang virus (MojPV) and representative paramyxoviruses from each of the other major genera within the *Paramyxovirinae* subfamily: Measles virus (MeV, Genbank AB016162.1), Newcastle disease virus (NDV, Genbank AF309418.1), Mumps virus (MuV, AB040874.1), and human parainfluenza virus 1 (HPIV-1, Genbank AF457102.1). NiV and HeV, as the prototypic members of *Henipavirus*, are highlighted in red. **(B)** HA-tagged matrix and C proteins were transfected in 293T cells as indicated. All the C proteins enhance the budding of their cognate M proteins, with the exception of “henipa-like” MojPV. Error bars represent standard deviations for 4 independent experiments. ns, not significant; *, p<0.05; **, p<0.01, 2-tailed Student’s t-test.

NiV, along with the closely related Hendra virus (HeV), are the founding members of the *Henipavirus* genus within the *Paramyxoviridae* family. Although sequencing of field samples from the natural bat reservoir suggests a wide diversity of henipaviruses, there are thus far only two additional complete or nearly complete sequenced henipavirus genomes: Cedar virus (CedPV) [38] and an African henipavirus isolated from bat in Ghana (Gh-M74a strain, GhV) [39] (Fig 7A). Another recently sequenced virus from rodents in China, Mojiang virus (MojPV) [40], has been suggested to be “henipa-like”, since it shares sequence similarity with the henipaviruses (Fig 7A) but has not yet been shown to share other conserved features, such as use of the ephrinB2 receptor.

Having identified a new role for the NiV C protein in budding, we wondered if this function might be conserved among the henipa- or henipa-like viruses. We co-transfected the C proteins of HeV, CedPV, GhV, and MojPV with their cognate matrix proteins (Fig 7B). The C proteins of HeV, CedPV, and GhV significantly enhanced M VLP release; by contrast, the C protein of MojPV did not enhance M VLP release. This result suggests that while C protein enhancement of budding is a feature conserved among known henipaviruses, it may not be conserved among more distantly related “henipa-like” viruses such as MojPV.

## Discussion

Although some enveloped viruses have been suggested to have budding mechanisms independent of the ESCRT pathway [3,41], such a mechanism has been described in molecular detail only for Influenza A virus, which encodes a factor capable of membrane scission [7]. Nipah virus was previously suggested to have an ESCRT-independent budding pathway, as NiV-M VLP budding in the absence of other virus components was insensitive to DN Vps4 inhibition [10].

**Fig 8:**
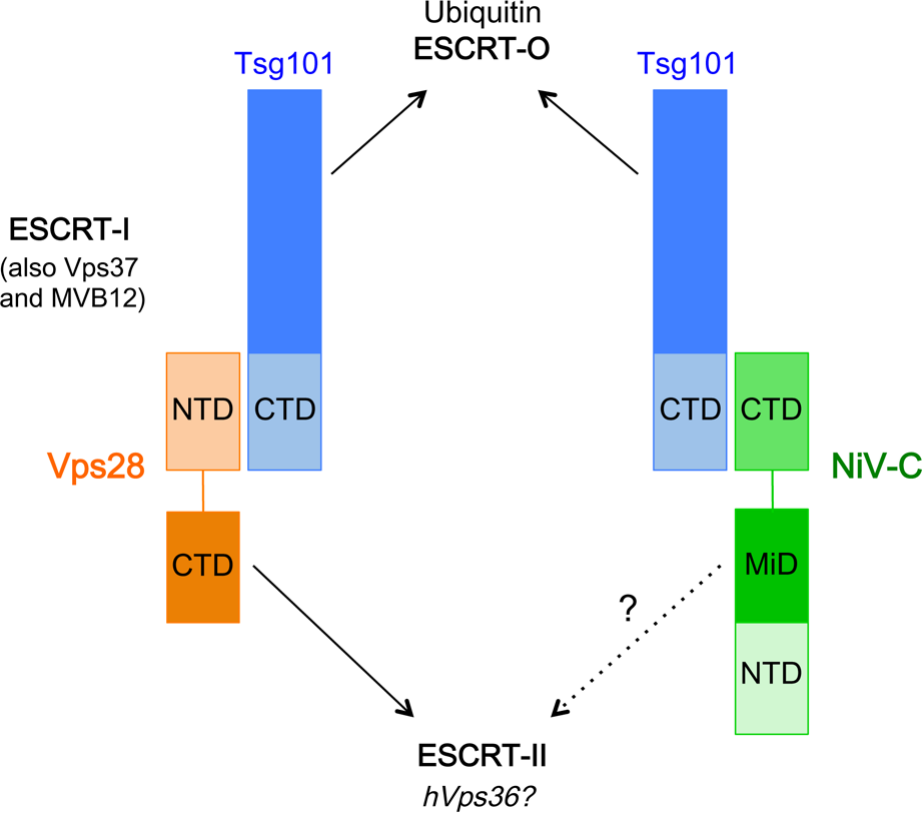
Model of NiV-C interactions with ESCRT. Vps28 acts as an adaptor between ESCRT-I and ESCRT-II. The NTD of Vps28 interacts with ESCRT-I via Tsg101-CTD, while the CTD of Vps28 interacts with ESCRT-II possibly via hVps36. The CTD of NiV-C also interacts with Tsg101-CTD, while the potential interaction of NiV-C-MiD with ESCRT-II or other downstream ESCRT factors remains to be defined.

In this work, we found that the efficient budding of live NiV is in fact sensitive to DN Vps4 inhibition (Fig 1), indicating an ESCRT-dependent budding pathway. This result also indicated that other viral component(s) besides NiV-M likely contribute to the budding process. Unexpectedly, we found that the viral C protein promotes budding by recruiting the ESCRT factor Tsg101, previously shown to play critical roles in the budding of other viruses [3,41]. The C-terminal domain of NiV-C interacted directly with the C-terminal domain of Tsg101 (Fig 5), and disruption of this interaction or specific depletion of Tsg101 resulted in abrogation of the budding enhancement function of NiV-C (Fig 6). We also found a suggestive sequence alignment between the middle domain of NiV-C and the C-terminal domain of the ESCRT factor hVps28 (Fig 4A). hVps28 plays an adaptor role in the ESCRT machinery, with one domain of hVps28 (the NTD) interacting with the ESCRT-I subcomplex via its essential component Tsg101, and the other domain of hVps28 (the CTD) interacting with a complex of downstream ESCRT factors including ESCRT-II components such as hVps36 [28,30]. The interesting parallel of one domain of NiV-C (the CTD), like hVps28-NTD, interacting with Tsg101-CTD while a different domain of NiV-C (the MiD) aligns to hVps28-CTD suggests that NiV-C-MiD, like hVps28-CTD, may also interact with downstream ESCRT factors (see model in Fig 8). Although we have not been able to detect hVps36 interaction with NiV-C, our finding that a cluster of residues conserved between NiV-C-MiD and hVps28-CTD was required for the NiV-C budding function (Fig 4C) supports the possibility that NiV-C may also act as an adaptor that interacts with multiple ESCRT components. The identity of potential downstream interacting factor(s) remains under investigation.

Our finding that NiV-C recruited ESCRT to mediate efficient budding was unexpected for several reasons. First, the C protein is the least conserved of the virus proteins in the *Paramyxovirinae* subfamily, with the C protein even being completely absent from the *Rubulavirus* and *Avulavirus* genera. Within the *Henipavirus* genus, only NiV-C and HeV-C are relatively similar (90% amino acid similarity) due to the high relatedness of NiV and HeV; the other pairwise comparisons between henipavirus C proteins show 39–52% similarity. That NiV-C should have such a critical function is therefore somewhat surprising. Second, the immune modulatory function of NiV-C has been well established, with multiple groups demonstrating clear effects of NiV-C on limiting type I interferon responses and proinflammatory cytokine upregulation [20–23]. NiV-C is the smallest of the viral proteins at 166 a.a., and if NiV-C embodied independent molecular mechanisms to both enhance budding and antagonize innate immunity, this would illuminate the principle that viruses have evolved to make the most of their limited coding capacity. It would be interesting to investigate, however, how much of the immune antagonism function of NiV-C is an indirect outcome of its recruitment of ESCRT. Viral recruitment of ESCRT factors may deplete their accessibility for normal cellular functions, and modulation of ESCRT would have pleiotropic effects due to the role of ESCRT in essential cell processes such as cytokinesis as well as multivesicular body formation, which is important for downregulation of transmembrane proteins via internalization into endosomes and subsequent degradation. Inhibition of ESCRT therefore leads to secondary effects including induction of autophagy, dysregulation of signaling pathways, and even modulation of RNAi activity, as RISC complexes are assembled on multivesicular bodies [42–44]. Third, the C protein is translated from an alternate reading frame within the NiV-P gene. There are thus strong coding constraints for the C protein resulting from the requirement to maintain the essential function of the NiV P protein [45]. That a virus protein under such coding constraints could mimic the sequence of a host factor, in this case the ESCRT factor Vps28, is remarkable. The extent to which the virus has used its limited coding capacity to recruit ESCRT for its budding pathway highlights its importance for virus replication and pathogenesis.

It is interesting to note that our results confirm that NiV-M can bud independently of ESCRT (Fig 2F), and it is possible that NiV-M possesses some intrinsic membrane deformation and scission capability, as reported for purified Newcastle disease virus matrix protein [46]. Nevertheless, recombinant C-deficient NiV, although replication-competent, is strongly attenuated in various cell lines *in vitro* as well as *in vivo* [19–22], including type I IFN-deficient Vero cells, indicating that there must be an unknown function of NiV-C independent of innate immune antagonism that is critical for virus replication. Our finding that NiV-C specifically recruits the ESCRT pathway to promote virus budding provides a mechanistic rationale for this heretofore unexplained phenomenon.

Finally, we found that the C proteins of other known henipaviruses also enhance matrix VLP release. A role in the budding pathway may therefore be a conserved feature of the henipavirus C proteins. We were interested to find that the C protein of Mojiang virus, which has thus far been considered “henipa-like” due to its greater sequence divergence (Fig 7A) [40,47], did not enhance budding. Further afield, the C proteins of some other paramyxoviruses such as certain members of *Respirovirus* have been thought to play a role in budding, although the data has been unclear or conflicting [12–15,48]. For example, for Sendai virus (SeV), the prototypic virus within *Respirovirus*, the C protein was suggested to enhance SeV-M-driven VLP budding through recruitment of the ESCRT component Alix [12,13]; in contrast, another study determined that knockdown of C protein expression or inhibition of ESCRT did not affect live SeV budding [15], thus leaving the significance of SeV-C for the budding process unclear. Other paramyxovirus C proteins have defined roles in viral RNA replication and antagonism of interferon signaling, and no known role in budding [45]. Within the henipaviruses, performing the same structural homology modeling search on HeV-C, CedPV-C, and GhV-C that was previously performed for NiV-C shows that while HeV-C also aligns to Vps28, due to the high sequence similarity between NiV-C and HeV-C, there appears to be no potential alignments of CedPV-C and GhV-C with factors associated with ESCRT. It will therefore be fascinating to determine whether the C-mediated enhancement of budding is ESCRT-dependent for the other henipaviruses, as it is for NiV.

Our finding that NiV-C recruits ESCRT to mediate efficient budding not only expands the universe of enveloped viruses that depend on this host pathway, but also defines a previously unknown, yet critical role for the henipavirus C proteins.

## Materials and Methods

### Cell lines

293T and Flp-In T-REx 293 cells (Invitrogen) were propagated in Dulbecco’s modified Eagle’s medium (Invitrogen) supplemented with 10% fetal bovine serum (Atlanta Biologicals) and penicillin/streptomycin. Flp-In T-REx 293 cells were additionally maintained in blasticidin and zeocin according to manufacturer protocol. To generate the Vps4A-inducible 293 cells, the Vps4A gene (the kind gift of Jiro Yasuda) was N-terminally tagged with HA and inserted into pcDNA5/FRT/TO (either wild-type Vps4A or containing the K173Q mutation that renders it dominant-negative), then transfected into the parental Flp-In T-REx 293 cells along with pOG44 (encoding the Flp-recombinase). Stable cell lines with doxycycline-inducible expression of wild-type or dominant-negative HA-Vps4A were generated by selection with hygromycin (replacing the zeocin) and blasticidin according to manufacturer recommendations. To generate the 293T cell line with destabilization domain (DD)-tagged endogenous Tsg101, the method was as described [36] but with several differences. The puromycin and blasticidin resistance donor constructs (as shown in Fig 6D) incorporated a left homology arm of 731 bp and a right homology arm of 536 bp specific to Tsg101. Further, a ecDHFR-based destabilization domain [49] sensitive to the compound trimethoprim (TMP) (Sigma, T7883) was used. The ecDHFR DD sequence was codon-optimized for human expression (Invitrogen) and synthesized as a GeneArt String (Invitrogen). Finally, a CRISPR construct with nickase (D10A) Cas9 was used, px335 (Addgene, cat# 42335, from Feng Zhang), with a double strand nick strategy for increased specificity. Upon single cell isolation and clonal expansion of the DD-Tsg101 cells, clones were genotyped with PCR using primers flanking (thus not contained within the donor constructs) the donor homology arms (as in Fig 6D and 6E). The sequence for the donor constructs is shown in S4 Fig, and sequences for CRISPR guide RNAs and primers are available on request. For virus-like particle budding and live virus infections performed with DD-Tsg101 cells, cells were incubated in either 10 uM TMP or DMSO vehicle for three days preceding the assay to ensure complete knockdown of DD-Tsg101. Removal of TMP was accomplished with three serial washes of trypsinized cells (pelleting, aspiration of supernatant, and resuspension) in media lacking TMP.

### Viruses and infections

Nipah virus (199901924 Malaysia prototype strain) was kindly provided by the Special Pathogens Branch (Centers for Disease Control and Prevention, Atlanta, GA, USA). The recombinant NiV expressing *Gaussia* luciferase (Gluc) is as previously described [16–18] and incorporates a Gluc-P2A-EGFP reporter as an independent ORF between the N and P genes. The NiV-C knockout virus was generated from the Gluc-expressing recombinant NiV as done previously [19,21], by mutating the two initiating start codons for NiV-C (t2429c, t2432c) as well as introducing a downstream stop codon (c2438a), all without disturbing the overlapping NiV-P ORF. For the Vps4A-inducible 293 infection, cells in 12-well were infected at a multiplicity of infection (MOI) of 2 for 45 minutes at 37°C, washed in the well (with the final wash saved for determination of residual background titers), and incubated with media with or without 16 ng/mL doxycycline. Supernatants were collected at the indicated time points and stored at -80°C. Titers were determined by plaque assay on Vero cells, and Gluc activity was detected with the BioLux *Gaussia* Luciferase Assay (New England Biolabs). The infection of DD-Tsg101 293T cells was similar, except with the cells in the presence or absence of trimethoprim (as described above) both preceding and after the infection. All work with live virus was carried out under biosafety level-4 conditions in the Robert E. Shope and the Galveston National Laboratory at the University of Texas Medical Branch.

### Plasmid constructs

The codon-optimized NiV-M gene in pcDNA3 (pNiV-M) is as previously described [50]. The codon-optimized NiV-F gene and NiV-G genes were as described [51] in pcDNA3 with C-terminal HA tags. The native sequences of NiV-N, -P, -V, -W, and -C were HA-tagged and amplified from the previously described pTM1-based expression constructs [52] (with NiV-V, -W and -C amplified from the P gene construct) and inserted into pcDNA3. The human Vps28 gene was synthesized with a N-terminal HA tag as a GeneArt String (Invitrogen) and inserted into pcDNA3. Tsg101 was amplified from pcGNM2/TSG-F (NIH AIDS Reagent Program, Cat# 11483, from Eric Freed) along with the additional N-terminal 10 residues of Tsg101 not present in the construct (MAVSESQLKK) and inserted into pCMV-3Tag-1A (Agilent), which appends a N-terminal 3XFLAG tag. FLAG-EGFP-Tsg101-CTD represents residues 303–390 of Tsg101 fused to the C-terminus of EGFP via a GSG linker in pCMV-3Tag-1A. The codon-optimized HeV-M gene [18] was inserted into pcDNA3 with a N-terminal HA tag via a double glycine linker. Sequences for CedPV-M (Genbank JQ001776.1), GhV-M (Genbank HQ660129.1), and MojPV-M (Genbank KF278639.1) were codon-optimized (Invitrogen), HA-tagged, synthesized as GeneArt Strings (Invitrogen), and inserted in pcDNA3. Likewise, sequences for NiV-C (Genbank AF212302.2), HeV-C (Genbank AF017149.3), CedPV-C, GhV-C, and MojPV-C were codon-optimized with C-terminal HA tags via a glycine linker, synthesized as GeneArt Strings (Invitrogen), and inserted in pcDNA3. The codon-optimized NiV-C-HA expression construct (pNiV-C-opt) was used as the basis for all NiV-C mutant constructs. HA-EGFP-C-CTD represents residues 128–166 of NiV-C fused to the C-terminus of HA-EGFP via a GSG linker in pcDNA3.

### Western immunoblotting

Samples in SDS Laemmli buffer were run on reducing SDS PAGE, either 10% Tris-glycine or Any kD TGX gels (Bio-Rad). Upon transfer to low fluorescence PVDF (Immobilon-FL, Millipore; or Immun-Blot LF, Bio-Rad), the membranes were incubated in Odyssey blocking buffer (Li-Cor Biosciences), then incubated with primary antibodies, followed by fluorescent secondary antibodies. For imaging on a Li-Cor Odyssey imaging system, the secondary antibodies were goat IRDye 800CW or 680LT antibodies (Li-Cor Biosciences). For imaging on a Bio-Rad ChemiDoc MP imaging system, the secondary antibodies were goat Alexa Fluor 647 or 546 antibodies (Invitrogen). The following primary antibodies were used: rabbit anti-NiV-M [50], rabbit anti-NiV-C (produced by 21st Century Biochemicals from rabbits immunized with a purified peptide corresponding to amino acids 13–31 of NiV-C), rabbit anti-HA (Novus, NB600-363), rabbit anti-COX IV (Li-cor Biosciences, 926–42214), mouse anti-Tsg101 clone 4A10 (Genetex, GTX70255), and mouse anti-FLAG clone M2 (Sigma, F3165).

### Virus-like particle budding assay

293T or Vps4A-inducible 293 cells in 6-wells were transfected with pNiV-M along with the indicated HA-tagged NiV protein expression plasmid, with pcDNA3 vector to 2 ug total per well, using BioT transfection reagent (Bioland) according to manufacturer protocol. The medium was changed at 4 hours post-transfection, and cell lysates and supernatants were collected at 24 hours post-transfection. Supernatants were clarified and ultracentrifuged through 20% sucrose at 145,000 x *g* for 1.5 hours. Pellets were resuspended directly in SDS Laemmli sample buffer. Serial dilutions of high-expressing sample were included on each gel during Western analysis to aid in quantification of relative amounts of NiV-M. The relative quantities were normalized to the NiV-M with vector only control, and the budding index was determined as (relative M in VLPs / relative M in cell lysates).

### Immunoprecipitation

293T cells in 6-wells were transfected with 1 ug of expression constructs or empty pcDNA3 vector for a total of 2 ug per well, using BioT transfection reagent (Bioland) according to manufacturer protocol. At 24 hours post-transfection, cells were lysed in cold lysis buffer (1% NP-40, 100 mM Tris-HCl pH 7.5, 150 mM NaCl, 5% glycerol, 1 mM EDTA, 1X protease inhibitor cocktail (Roche)), clarified at 14k rpm for 5 minutes at 4°C, and incubated with 3 ug of rabbit anti-NiV-M [50] or rabbit anti-HA (Novus, NB600-363) at 4°C overnight. Following incubation with protein G agarose (Pierce) for 2 hours at 4°C, the bound protein was washed 4 times with cold wash buffer (same composition as lysis buffer but with 0.2% NP-40), then eluted in reducing Laemmli SDS sample buffer at 95°C.

### Confocal microscopy

pNiV-M and pNiV-C-opt were co-transfected into 293T cells plated on coverslips coated sequentially with poly-L-lysine and collagen. At 24 hours post-transfection, cells were fixed in 2% paraformaldehyde, washed 3 times with PBS, incubated in blocking buffer (0.5% saponin, 3% BSA in PBS), then incubated with 4.7 ug/mL rabbit anti-NiV-M [50] and 4 ug/mL chicken anti-HA (Novus, NB600-361) in blocking buffer. After 3 washes in 0.5% saponin in PBS, the coverslips were incubated with goat anti-rabbit Alexa Fluor 594 and goat anti-chicken Alexa Fluor 488 (1:1000, Invitrogen). After further washing and staining with DAPI to visualize nuclei, coverslips were mounted on slides. Confocal imaging was performed on a Leica SP5 confocal microscope in the microscopy core facility at the Icahn School of Medicine at Mount Sinai.

### Recombinant protein purification

FLAG-EGFP and FLAG-EGFP-Tsg101-CTD (both with the A206K mutation in EGFP that renders it monomeric), as well as full length NiV-C (native sequence) with a N-terminal HA tag, were inserted into the pET-15b vector (Novagen), which appends an additional N-terminal 6XHis tag. The recombinant proteins were purified from BL21(DE3) *E*. *coli* as previously described [36] with minor modifications. Cells at OD_600_ 0.5 were induced with 0.5 mM IPTG at 28°C for 4 hours, then collected and lysed in sodium phosphate buffer (pH 8) containing 10 mM imidazole. 6XHis-tagged proteins were purified on 5 mL HisTrap HP (GE Healthcare) columns with increasing imidazole concentrations up to 500 mM, with 0.1% triton X-100 present throughout to maintain protein solubility. Pure fractions were dialyzed overnight into PBS with 5% glycerol and 0.1% triton X-100.

### Transmission electron microscopy (TEM)

DD-Tsg101 293T cells in the presence or absence of TMP were infected with wild-type NiV at a MOI of 2 as described above. At 24 hours post-infection, cells were fixed with PFGPA solution (2.5% formaldehyde, 0.1% glutaraldehyde in 0.05 M cocadylate buffer pH 7.3, 0.01% picric acid and 0.03% CaCl_2_) for 24 hours at 4°C. Cells were collected and post-fixed in 1% OsO_4_ in 0.1 M cocadylate buffer for 1 hour at room temperature. Cells were then *en bloc* stained with 2% aqueous uranyl acetate (UA) for 20 minutes at 60°C, and dehydrated through a graded series of ethanols. Samples were then processed for infiltration using mixtures of propylene oxide and epoxy (poly/bed 812; Polysciences Inc) and embedded in 100% poly/bed 812. After overnight polymerization at 60°C, ultrathin sections were cut on Leica EM UC7 ultramicrotome, stained with lead citrate and examined in a Philips 201 transmission electron microscope at 60 kV. For immunogold staining, cells were stained *en bloc* with 2% UA without prior post-fixation, dehydrated in ethanol, and embedded in LR White resin medium grade (Electron Microscopy Sciences). Ultrathin sections were stained with anti-NiV-N antibody (kindly provided by Christopher Broder, Uniformed Services University) followed by secondary goat anti-rabbit IgG conjugated to 15 nm colloidal gold particles (Electron Microscopy Sciences). Sections were fixed with 2% aqueous glutaraldehyde, and stained with 2% UA and lead citrate.

## Supporting Information

S1 Fig Vps4A-inducible cells uniformly overexpress WT or DN Vps4A upon induction. See Materials and Methods for details on cell line creation. *Top panel*, WT and DN Vps4A-inducible 293 cell lines were induced with 16 ng/mL doxycycline. At 24 hours post-induction, parental Flp-In T-REx 293 cells and uninduced vs. induced Vps4A cells were fixed, permeabilized, and stained with anti-HA-PE (Miltenyi) to detect intracellular HA-Vps4A. Induction with doxycycline results in uniform overexpression of WT or DN Vps4A. *Bottom panel*, time course of induction showing that HA-Vps4A overexpression is detectable at least by 6 hours post-induction.
(PDF)

S2 Fig Expression levels of NiV-M and NiV-C from transfection are within the natural levels from NiV infection. 293T cells were transfected with NiV-M and NiV-C-HA as in the budding assay in Fig 2B, or infected with wild-type NiV at MOI 2. Cell lysates were collected at 24 hpi, and Western analysis with anti-NiV-M or anti-NiV-C showed that NiV-M and NiV-C expression levels from transfection are within the levels from infection. The HA tag on the transfected NiV-C-HA results in an upward molecular weight shift from the native C protein.
(PDF)

S3 Fig Destabilization of DD-Tsg101 results in impaired cellular growth. 2 x 10^4^ DD-Tsg101 293T cells per well were plated from a master mix cell suspension into either DMSO vehicle- or TMP-containing media in 6-well plates. Starting 1 day post-plating and each day following, cells were trypsinized and counted. The media was changed on day 3 for cells to be collected on following days. Error bars represent standard deviations for 3 replicates. Cell counts became significantly different between +/- TMP starting at 4 days post-plating. ***, p<0.001, two-way ANOVA followed by Bonferroni posttests. Parental WT 293T cells in the same conditions showed no significant difference between +/- TMP at any day over the 6 day time course.
(PDF)

S4 Fig Annotated sequence of Tsg101 HDR donor constructs to knock-in a destabilization domain tag onto the N-terminus of endogenous Tsg101. (PDF)
