# Supplementary figures and images for: Nipah Virus C Protein Recruits Tsg101 to Promote the Efficient Release of Virus in an ESCRT-Dependent Pathway
Source: PLoS Pathog. 2016 May 20;12(5):e1005659. doi: 10.1371/journal.ppat.1005659 (PMC4874542; doi:10.1371/journal.ppat.1005659)

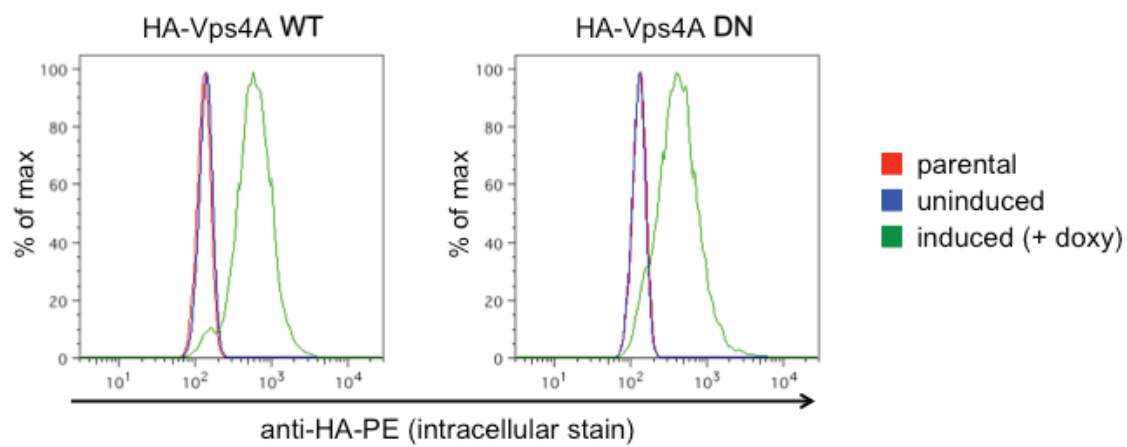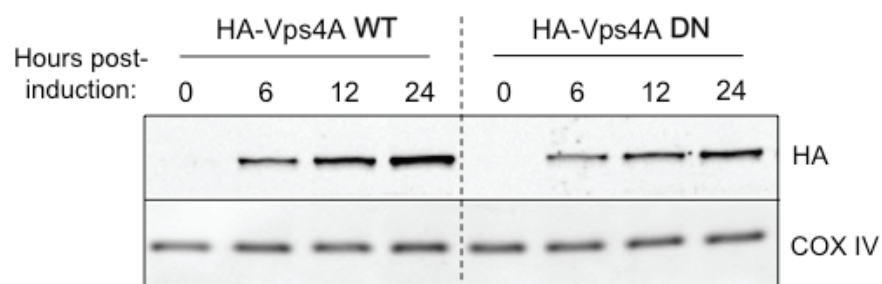

Supplement: S1 Fig — See Materials and Methods for details on cell line creation. Top panel, WT and DN Vps4A-inducible 293 cell lines were induced with 16 ng/mL doxycycline. At 24 hours post-induction, parental Flp-In T-REx 293 cells and uninduced vs. induced Vps4A cells were fixed, permeabilized, and stained with anti-HA-PE (Miltenyi) to detect intracellular HA-Vps4A. Induction with doxycycline results in uniform overexpression of WT or DN Vps4A. Bottom panel, time course of induction showing that HA-Vps4A overexpression is detectable at least by 6 hours post-induction. (PDF) [file ppat.1005659.s001.pdf]

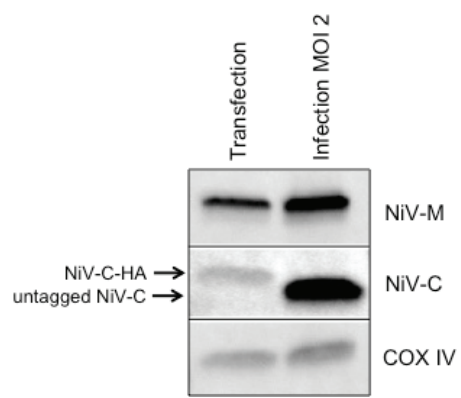

Supplement: S2 Fig — 293T cells were transfected with NiV-M and NiV-C-HA as in the budding assay in Fig 2B, or infected with wild-type NiV at MOI 2. Cell lysates were collected at 24 hpi, and Western analysis with anti-NiV-M or anti-NiV-C showed that NiV-M and NiV-C expression levels from transfection are within the levels from infection. The HA tag on the transfected NiV-C-HA results in an upward molecular weight shift from the native C protein. (PDF) [file ppat.1005659.s002.pdf]

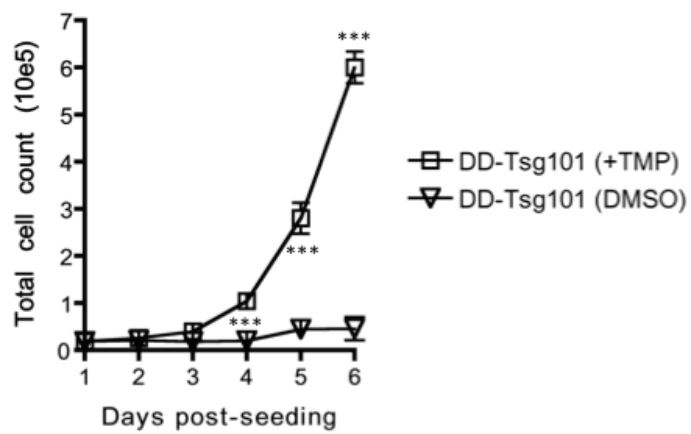

Supplement: S3 Fig — 2 x 10^4^ DD-Tsg101 293T cells per well were plated from a master mix cell suspension into either DMSO vehicle- or TMP-containing media in 6-well plates. Starting 1 day post-plating and each day following, cells were trypsinized and counted. The media was changed on day 3 for cells to be collected on following days. Error bars represent standard deviations for 3 replicates. Cell counts became significantly different between +/- TMP starting at 4 days post-plating. ***, p<0.001, two-way ANOVA followed by Bonferroni posttests. Parental WT 293T cells in the same conditions showed no significant difference between +/- TMP at any day over the 6 day time course. (PDF) [file ppat.1005659.s003.pdf]
