# Supplementary material for: Nipah Virus C Protein Recruits Tsg101 to Promote the Efficient Release of Virus in an ESCRT-Dependent Pathway
Source: PLoS Pathog. 2016 May 20;12(5):e1005659. doi: 10.1371/journal.ppat.1005659 (PMC4874542; doi:10.1371/journal.ppat.1005659)
Supplement: S4 Fig — (PDF) [file ppat.1005659.s004.pdf]

The following donor insert was inserted into the PCR2.1 vector (Invitrogen).

KpnI and EcoRI restriction sites

Homology arms (all underlined sequence, regardless of color)

TCOF1 exon 1

Puromycin resistance (alternative Blast^{R} is below)

GSG linker

P2A ribosomal skipping sequence

Destabilization domain

3XGGGGS linker

First residue of Tsg101

GGTACC

CATTTATGGGTGGGTACAGAGGAGAAATTCTGACTTACGGAATGATTTCCTGGAGAAGAAATTACTATCTGGCTT
CTTGTGAAACAAAGGCTCTACACTTTACCCTTGCCAACGTATAGTATGAAGGTCTTTAAGCTTAAAAAAAATCAT
TAATTAGAGGACCACTGGGCATCATCTAAGGCAAGTGTATGACTTTAGAGTCGGGAGAAATTTGCGCCTGAGCTA
GAAATGCACTAGCCTACCATTATCACAAGAGCAAGTCAGTGACAGAACAGTTCTCTGACCGCCAGTGCAGTGCCC
TCCCACTTGATCAATCAGTCATTAAGGCTCACTGGCAAGCCCTTCGTACTTCTTCCCTCTCCGGCACTAGCTTCC
TTAAGTCGACTCTGTGAAATACCTGCTAATCTTATCCTGAACCAGAGTTTCGACCCTCGGAACAGTCTTGCCCAC
ATCTTTGGGACCCGATTCGTCCTGCTGATTCCGGCACTCGAGGGGCGGGTTGGTTGGAACCCACCCCACCAGGCC
CCTCTCAATCCCACACGGTGTCCACCCTTGCCCGCAGGACGGCGGCCCGGAAGTGACGTAGTGGAAGC**GGAAGTG**
GTGTAGTGGTGCCGACTTCCTGTTGTTTGAGGCCGGGTTGGGGGTGTGCGATTGTGTGGGACGGTCTGGGGCAGC
CCAGCAGCGGCTGACCCTCTGCCTGCGGGGAAGGGAGTCGCCAGGCGGCCGTC

ATGACCGAGTACAAGCCCACGGTGCGCCTCGCCACCCGCGACGACGTCCCCAGGGCCGTACGCACCCTCGCCGCC
GCGTTCGCCGACTACCCCGCCACGCGCCACACCGTCGATCCGGACCGCCACATCGAGCGGGTCACCGAGCTGCAA
GAACTCTTCCTCACGCGCGTCGGGCTCGACATCGGCAAGGTGTGGGTCGCGGACGACGGCGCCGCGGTGGCGGTC
TGGACCACGCCGGAGAGCGTCGAAGCGGGGGCGGTGTTCGCCGAGATCGGCCCGCGCATGGCCGAGTTGAGCGGT
TCCCGGCTGGCCGCGCAGCAACAGATGGAAGGCCTCCTGGCGCCGCACCGGCCCAAGGAGCCCGCGTGGTTCCTG
GCCACCGTCGGCGTCTCGCCCGACCACCAGGGCAAGGGTCTGGGCAGCGCCGTCGTGCTCCCCGGAGTGGAGGCG
GCCGAGCGCGCCGGGGTGCCCGCCTTCCTGGAGACCTCCGCGCCCCGCAACCTCCCCTTCTACGAGCGGCTCGGC
TTCACCGTCACCGCCGACGTCGAGGTGCCCGAAGGACCGCGCACCTGGTGCATGACCCGCAAGCCCGGTGCC

GGATCCGGA

GCCACGAACTTCTCTCTGTTAAAGCAAGCAGGAGACGTGGAAGAAAACCCCGGTCCC

ATGATCAGCCTGATTGCCGCCCTGGCCGTGGACTACGTGATCGGCATGGAAAACGCCATGCCCTGGAACCTGCCT
GCCGACCTGGCCTGGTTCAAGCGGAACACCCTGAACAAGCCCGTGATCATGGGCCGGCACACCTGGGAGTCTATC
GGCAGACCTCTGCCCGGCAGAAAGAACATCATCCTGAGCAGCCAGCCCAGCACCGACGACAGAGTGACCTGGGTC
AAGAGCGTGGACGAGGCCATTGCCGCCTGCGGAGATGTGCCTGAGATCATGGTCATCGGCGGAGGCAGAGTGATC
GAGCAGTTCCTGCCCAAGGCCCAGAAGCTGTACCTGACCCACATCGACGCCGAGGTGGAAGGCGACACCCACTTC
CCCGACTACGAGCCCGATGACTGGGAGAGCGTGTTCAGCGAGTTCCACGACGCCGACGCCCAGAACAGCCACAGC
TACTGCTTCGAGATCCTGGAACGGCGG

GGT GGA GGT GGA TCT GGT GGA GGT GGA TCT GGT GGC GGC GGT TCA

GCGGTGTCGGAGAGCCAGCTCAAGAAAATGGTGTCCAAGGTGAGGCTGCGACGCGCTCGCCTCCCAGGGCGCGCC
CACCGCTCCCTTCCGCGCCCTGTCGAGTCCGTCCCGGCCCAGCCAAGCAAGCTTCCCAGACGGGCCGGAAGCCCC
GGTGCAGTCCTTAGCGACCTCCTCAGAACCCCGCCCCGAGGCGCCTGTCGCCTGGTGCGGGAATCCCCGTACGGG
AGCTGGGAGGGTGGGGGACGGCGACAGTCAACAAAGGCGTGGAGCGGAGGCTACCTGACACCTGCCGCCCACCCG
CCCTCCTCTCTTCCACTGAGTTTGGAGCTGTCTGTGGGGCAGTGTAGTTTTTCGTTTGTTTTTATAAACACAAAC
AAGGGATCATACTTAGTTGTAGATCTGAGGCAATCCTCTACTCCTGTCCCAATCTAGGGATCCTCCAGTCTTAGC
CTTTCTTTTGCCGACCCGCAGAGAGTGTGGCCTTTTCAGTTTTGTATTCTCCAGAAAAGAAGCGAAAATACCAGT
CCCTTACTGCC

GAATTC

Blasticidin resistance:

ATGGCCAAGCCTTTGTCTCAAGAAGAATCCACCCTCATTGAAAGAGCAACGGCTACAATCAACAGCATCCCCATC
TCTGAAGACTACAGCGTCGCCAGCGCAGCTCTCTCTAGCGACGGCCGCATCTTCACTGGTGTCAATGTATATCAT
TTTACTGGGGGACCTTGTGCAGAACTCGTGGTGCTGGGCACTGCTGCTGCTGCGGCAGCTGGCAACCTGACTTGT
ATCGTCGCGATCGGAAATGAGAACAGGGGCATCTTGAGCCCCTGCGGACGGTGCCGACAGGTGCTTCTCGATCTG
CATCCTGGGATCAAAGCCATAGTGAAGGACAGTGATGGACAGCCGACGGCAGTTGGGATTCGTGAATTGCTGCCC
TCTGGTTATGTGTGGGAGGGC
